# Wayfinding with Impaired Vision: Preferences for Cues, Strategies, and Aids (Part II—Perspectives from Orientation and Mobility Instructors)

**DOI:** 10.3390/brainsci16010006

**Published:** 2025-12-20

**Authors:** Dominique P. H. Blokland, Maartje J. E. van Loef, Nathan van der Stoep, Albert Postma, Krista E. Overvliet

**Affiliations:** 1 Experimental Psychology, Helmholtz Institute, Utrecht University, 3584 CS Utrecht, The Netherlandsa.postma@uu.nl (A.P.); k.e.overvliet@uu.nl (K.E.O.); 2 Human Machine Teaming, Defense, Safety, and Security, Nederlandse Organisatie voor Toegepast-Natuurwetenschappelijk Onderzoek (TNO), 3769 DE Soesterberg, The Netherlands; nathan.vanderstoep@tno.nl

**Keywords:** orientation and mobility training, O&M, rehabilitation, individual differences, white cane, guide dog, route learning, visual impairment, low vision, blindness

## Abstract

Background/Objectives: People with visual impairments can participate in orientation and mobility (O&M) training to learn how to navigate to their desired destinations. Instructors adapt their approach to each individual client. However, assessments of client characteristics and resulting instructional adaptations are not standardised and may therefore vary. This study aimed to identify which individual differences instructors consider during O&M training and why. Methods: We conducted semi-structured qualitative interviews with 10 O&M instructors. Participants were asked to describe how they prepare for a training trajectory, and to describe a route they taught a specific client. Thematic analysis was used to determine instructional choices and the relevant client-specific factors. Results: We observed a common four-step instructional process in which clients are taught to notice, interpret, act upon, and anticipate relevant sensory cues until a destination is reached. Four main themes captured the individual differences impacting this process: Sensory modalities, Capacities and limits, Personal contextual characteristics, and Training approach. Conclusions: Instructors perceive route learning to be shaped by clients’ sensory abilities (even fluctuating within sensory modalities), mental and physical capacities (especially concentration and energy), and personal characteristics (especially age and anxiety). The dynamic social context in which training takes place (e.g., the instructor–client relationship) is shaped by individual differences between both clients and instructors. We speculate that trust-related themes (e.g., building confidence) may explain why certain client characteristics are emphasised by instructors, as they are associated with training outcomes.

## 1. Introduction

Navigation is a challenging undertaking for people with visual impairments (VIPs) [1,2,3,4,5,6,7,8,9]. To address these challenges, VIPs can request individualised Orientation and Mobility (O&M) training from professionally trained O&M specialists [10,11]. The goal of O&M training is to increase the capacity of VIPs to navigate autonomously, efficiently, and safely to their desired destinations [10,11]. In context of O&M, orientation is defined as the general ability to understand one’s own spatial location within an environment [10]. This encompasses skills like spatial updating and the use of spatial knowledge such as survey knowledge, route knowledge, landmark knowledge, and relative spatial relationships like distances and directions [12,13]. Mobility refers to the ability and facility of movement necessary for travelling between locations [10,13]. Key mobility skills include obstacle avoidance, managing tripping hazards, and noticing slopes and changes in walking surface texture [2,12,14]. For successful orientation and mobility, VIPs need to learn how to use non-visual sources of information to navigate, though which information they prefer using and how varies per person. In a related study (part I, [2]), we examined which information VIPs focus on during O&M, and why and how they report using it, with particular attention to their preferred information sources and the personal and environmental factors that are relevant for these preferences. As these wayfinding preferences and strategies may vary between VIPs, O&M instructors take into account their visually impaired clients’ personal preferences during O&M training based on their professional expertise and experience. Though, which factors instructors focus on and how they incorporate them into their instructional practice may vary across instructors. The current paper provides an in-depth overview concerning which individual differences are relevant for O&M instructors, for which reasons, and how they shape instructors’ training strategies in practice.

During the intake and the initial O&M training session, the O&M instructor and the client discuss desired destinations, after which the specific routes to and from these locations are planned by the instructor. Subsequent sessions consist of practicing these routes, usually with an emphasis on proper cane techniques [11,15,16,17]. Orientation and mobility skills are typically taught from an egocentric perspective (i.e., in a route-like, body-centred, turn-by-turn manner from the viewpoint of the traveller). Instructors commonly teach anchor-point strategies, where clients learn a certain route by following sequential landmarks, and route strategies (also referred to as response strategies), where clients learn a certain route by memorising a fixed sequence of actions associated with landmarks or other environmental features [18,19,20,21,22]. The utility of these strategies also becomes evident in the way VIPs themselves describe their routes (Blokland et al. [2]). These reports often involve landmark-to-landmark route descriptions, including the use of maintenance landmarks, such as using a particular wall according to a shorelining method until encountering their next reference point (also called ‘walltrailing’, [23]).

To effectively plan the desired routes, the O&M instructor must determine which cues, landmarks, paths, and turns would be most fitting and safe for a particular individual. These choices not only depend on the mental and physical abilities of the client and the specific type, severity, and onset of the visual impairment, but also on subjective factors. Namely, the O&M instructor assesses their clients’ mobility wants and needs, as well as the type of sensory cues and navigational strategies they prefer using [11,24]. The instructors’ assessments of these individual differences are partially based on clients’ self-report and partially on instructors’ own professional judgement of a client’s behaviour and remarks. As a result, the assessment of an individual client’s subjective preferences is in itself subjective, shaped by each instructor’s own priorities, prior experiences, and expertise.

Prior studies primarily have relied on VIPs’ self-reports, which, while valuable, do not capture broader patterns that describe VIPs’ wayfinding processes in practice. Yet an underexplored source of insight comes from O&M instructors, who are uniquely positioned to provide expert insight into the complex, individualised process of wayfinding with low vision: they directly observe how variations in sensory abilities, personal characteristics, and contextual factors shape clients’ everyday mobility. Trainers may pick up signals and behaviours of which their clients themselves are unaware. Despite this clear relevance of taking the O&M instructors’ perspective into account, no existing research has, to our knowledge, examined in depth how O&M instructors recognise, interpret, and incorporate these individual differences into their instructional decision-making. Instructors work with a wide range of visually impaired clients across diverse settings, so their observations can reveal patterns that extend beyond individual reports. This study therefore adds to the existing body of knowledge by providing the first systematic, qualitative analysis of O&M instructors’ perspectives on individual differences in wayfinding with low vision. We identify how instructors conceptualise and respond to individual differences among their clients within the context of their instructional practices, with a specific focus on personal characteristics and the use of sensory information during route-learning. This offers a novel, practice-grounded account of the factors that meaningfully influence route-learning experiences and everyday wayfinding for visually impaired individuals.

To this end, we conducted semi-structured qualitative interviews with ten O&M instructors with varying levels of experience with mobility training from different organisations and foundations within the Netherlands. We asked participants to describe how and why they chose a certain route for a particular client. Thematic analysis was performed to formulate themes describing which personal, environmental, and contextual factors are relevant to take into account in context of route-learning while visually impaired according to O&M specialists.

## 2. Materials and Methods

### 2.1. Sample

In total, ten Orientation and Mobility (O&M) instructors participated in the study (7 women, 3 men), mean age 42.5 ± 13.7 years. Experience as O&M instructor ranged from two to 40 years. Participants were recruited via a written advertisement distributed within collaborating organisations. Mobility instructors from all three mobility training providers within the Netherlands were recruited (i.e., Royal Dutch Visio, Bartiméus, and De Robert Coppes Stichting). Participants received compensation insofar as the hours spent on the study could be logged as paid working hours with their employer. See Table 1 for an overview of the sample descriptives. There were no exclusion criteria, all interested individuals were able to participate in the study. Interested participants were informed and asked to provide written consent according to protocols approved by the Faculty Ethics Review Board of the Faculty of Social and Behavioural Sciences at Utrecht University (protocol code 23-0279, approved 1 January 2023).

### 2.2. Design of the Interview Protocol

Face-to-face, semi-structured, qualitative interviews were held with the O&M instructors. A methodological approach was used similar to the methodology described by Blokland et al. [2]. The initial interview protocol was constructed to include introductory questions, transition questions, key questions, and closing questions [25,26,27]. Key questions were formulated to ask participants about their experiences during O&M training sessions concerning their clients’ use of sensory information during route learning. The main questions of the protocol are ‘How do you prepare for the first training session with a new client?’, in which follow-up questions are included concerning both the characteristics of the client and of the route, and ‘Could you describe the route that the client wanted to learn, from front door to destination?’, in which follow-up questions are included concerning the specific landmarks chosen and the participant’s rationale behind choosing these landmarks. The initial protocol was assessed by the research team according to Braun and Clarke’s [27] quality assurance checks, and tested in a pilot study. The final protocol can be found in Appendix A.

### 2.3. Procedure

Interested participants reached out to the research team via e-mail, in response to which they received a written information letter about the study. In case of continued interest, they were asked to provide written consent and a time and place for the interview was agreed upon. Interviews were held in an office space of a collaborating organisation, in a restaurant, or at a participant’s home. Each interview appointment lasted approximately one hour. At the start of the interview appointment, the participant would be once again briefed about the goal and the background of the study, and questions were answered when applicable. The interview itself followed the protocol as described in Appendix A. Interviews were recorded on two audio recorders (Olympus WS-853, and Olympus WS-852 as a back-up) resulting in two audio recordings per interview. Towards the end of the appointment, participants were thanked for their participation and debriefed about the course of events regarding data analysis.

### 2.4. Data Analysis

#### 2.4.1. Pre-Processing

For each interview, the best-quality audio recording was converted to text using speech-to-text software (Amberscript, [28]). Corrections to the transcripts were made manually by author DB or ML according to an intelligent verbatim approach [29]. Identifiable information was anonymised. The corrected, anonymised transcripts were used for analysis.

#### 2.4.2. Thematic Analysis

Transcripts were analysed using thematic analysis [30]. The goal of the analysis was to develop themes that describe observed meaningful patterns within the data. Analysis was carried out in NVivo (v14.23.1, [31]). Author ML functioned as the main coder during the coding process. Inductive and semantic coding was used to label the data [27], though codes were partially inspired by those used in Blokland and colleagues [2]. In total, four coding rounds were carried out.

The first coding round consisted of thorough re-reading of the interview transcripts (author DB and author ML), and providing all text fragments with initial codes (ML). Codes were discussed amongst authors ML and DB until agreement was reached about reformulation or reattribution of existing codes, or the formulation of new codes. Coding round one ended with all text fragments of each dataset being provided with codes.

During the second coding round, all initial codes were evaluated. Codes were either split up when they consisted of multiple meaningful elements, merged when they consisted of similar meaningful elements, removed when deemed uninformative for answering the research questions, or preserved when none of these criteria were met. After this step was completed, all data were re-evaluated and the updated, new, or existing codes were added or removed when appropriate. At the end of round two, an initial analysis of the associative and hierarchical relationships between codes was carried out as to group them into candidate themes. Multiple discussion sessions were held in which authors DB, KO, or AP asked questions about the meaning, motivation, and relevance of candidate themes until agreement was reached. Codes were adjusted accordingly. The second coding round ended when the first coder judged that all data were accounted for by the current codes and a first version of potential thematic map was formed.

The third coding round started with second coder DB applying the existing codes to samples of two interviews [32]. The sample fragments were picked by main coder ML according to estimated information density, with the assumption that the most code-dense fragments would elucidate possible disagreement between raters most efficiently. Interrater reliability was calculated via the coding comparison query in NVivo [31,32,33]. For codes with an observed inter-rater reliability lower than a Cohen’s kappa coefficient of 0.4, main coder ML and second coder DB would discuss and revise the code names and code descriptions. Authors ML and DB would repeatedly re-evaluate potential main themes and relations between (sub-)themes using author DB’s findings during inter-rating as a starting point. The third coding round ended when a Cohen’s kappa coefficient of 0.4 or larger was observed for all inter-rated codes, an updated version of the thematic map was agreed upon, and saturation was reached. Saturation was evaluated using a code meaning approach, whereby successive interviews were examined for new dimensions or nuances within existing codes. Saturation was considered reached when no further conceptual elaboration could be made.

The fourth coding round consisted of re-evaluation of the thematic map by refining candidate themes, assessing hierarchical relationships between (sub-)themes, and the grouping of sub-themes. To this end, author ML carried out multiple coding comparison queries in NVivo to find meaningful associations between (sub-)themes. Firstly, coding comparison queries were carried out to find out how often certain subsets of codes were mentioned by each participant (e.g., concerning different factors of O&M training). Secondly, coding comparison queries were carried out to find out how often certain codes co-appeared (e.g., use of sensory cues in different contexts). Findings were used by authors ML and DB to update the thematic map as to form a coherent map consisting of large overarching themes that capture the data. At the end of coding round four, the thematic map was discussed by authors AP, KO and DB, and presented to a focus group consisting of two visually impaired individuals and three O&M instructors that were recruited via collaborating organisations. The disagreements and clarifications that resulted from the discussions within the research team as well as the focus group’s input were used to further refine the themes and their relationships. The fourth coding round ended after consensus was reached on the formulation of and relationships between themes within the thematic map.

#### 2.4.3. Theme Development and Thematic Map Design

The thematic map is the main outcome of the study. It was construed based on data-driven decisions. The main factors in its construction were how often our participants brought up certain topics, which topics were often discussed in the same text fragment, and why participants brought up these topics. These factors were used, respectively, to determine which codes to include in the map, which associations between codes or (sub-)themes- to add, and which sub-themes could be grouped into overarching sub-themes.

### 2.5. Focus Group

The main themes and sub-themes were presented to a focus group consisting of three O&M instructors and two experts by experience. The focus group contributed two points of interest. First, there was consensus within the focus group that the type of environment (e.g., rural, village, city, or busy city) strongly influences O&M training, route learning, and choice of place of residence for people with visual impairments, and that this would be a suitable finding for the ‘Priorities and Lifestyle’ sub-theme. However, as residential area did not emerge as a sub-theme from our analysis due to a lack of discussion on this topic during the interviews themselves, residential area is not included as a sub-theme in the thematic map. Second, trust was presented to the focus group as a factor that connects themes and could serve as an explanation for why O&M instructors discussed certain individual differences. The focus group emphasised that sometimes trust, e.g., building a client’s self-confidence, might be a goal in itself, regardless of the acquisition of the more technical skills. In this way, O&M training might still be seen as successful if a client has gained more trust in their own independent wayfinding skills, even though the official goal of independent route-following is not achieved.

## 3. Results

**Figure 1:**
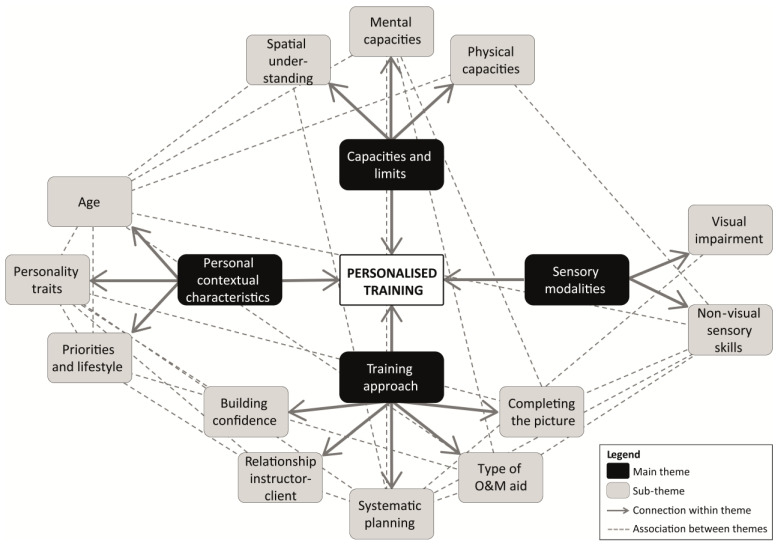
Thematic map. This figure shows all themes generated in this study and their relations. Four main themes were created (black boxes), each with two to five sub-themes (grey boxes). Arrows between themes indicate connections between themes. The arrows from the main themes towards the goal of personalised training represent the idea that the main themes inform the personalization of the training. Arrows from the main themes towards the sub-themes represent the idea that the sub-themes are different components from the main theme. Dotted lines indicate an associative relationship between sub-themes from different main themes.

**Figure 2:**
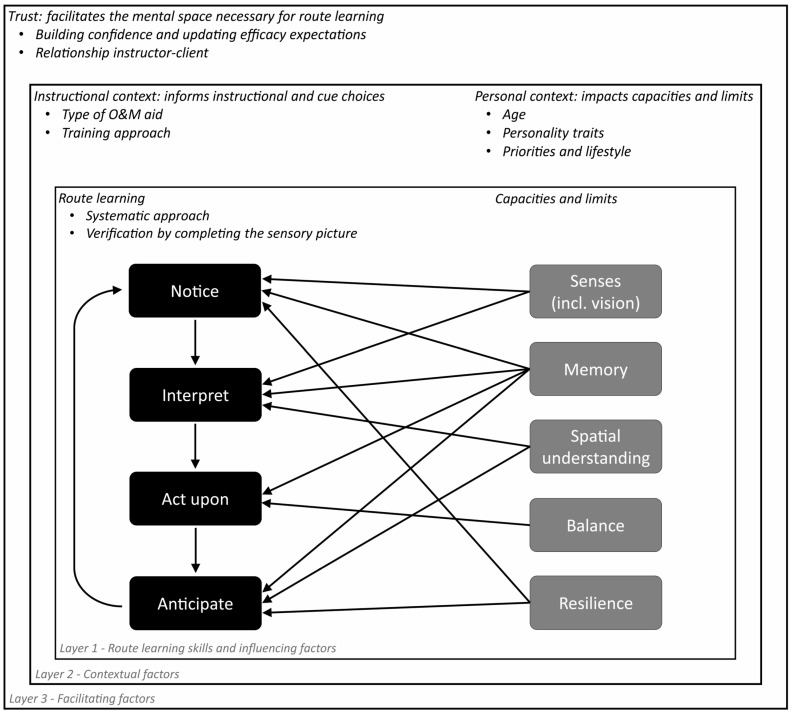
Conceptual model of how themes might dynamically interact. This figure displays several layers of individual differences relevant in context of route learning during O&M training. The route learning process was found to involve four key steps surrounding sensory cue use (shown here in black) that are repeated until a destination is reached, as described by the ‘Systematic Planning’ sub-theme. The arrows between these steps indicate the order in which the steps are typically carried out. All other themes were found to be directly or indirectly associated with variations in skill for each of these steps. The innermost layer represents the steps of the route learning process itself, as well as capacities and limits that were reported to directly impact route learning skill (in grey). The arrows between these factors on the one hand and the steps taken during route-following on the other hand indicate which factors impact which steps. The middle layer represents themes related to the instructional and personal context of the training, which include factors that were associated with route learning more indirectly. The outer layer represents sub-themes related to trust, which include factors that facilitate the route learning process by creating the necessary mental space (or ‘peace of mind’) to safely and independently navigate in the first place.

We carried out a thematic analysis of the qualitative interview data with Orientation and Mobility (O&M) instructors. The result of this analysis is the formulation of four main themes with two to five sub-themes describing which individual differences between visually impaired individuals (VIPs) are relevant in context of O&M. Whereas Figure 1 provides a descriptive overview of the main themes (i.e., displaying which themes are associated), Figure 2 provides an interpretative overview of the relations between the main themes in context of O&M training (i.e., focusing on how these themes are associated). We describe how training generally seems to follow a four-aspect structure (i.e., teaching a VIP strategies to notice, interpret, act upon, and anticipate relevant sensory cues), and that individual differences between clients are relevant for each step of this process. That is, the instructors’ Training approach (first theme) depends on their assessment of a specific client’s Personal contextual characteristics (second theme), Sensory modalities (third theme), and Capacities and limits (fourth theme).

### 3.1. Training Approach

The goal of O&M training is always to make sure the client can travel safely and independently to their chosen target locations. The steps that need to be learned here are being able to notice, interpret, act upon, and anticipate sensory cues along a route. Nevertheless, the instructor’s strategy for achieving these goals varies per instructor and per client. In this regard, it matters to O&M instructors how well a client can ‘Systematically Plan’ out their routes and ‘Complete Their Sensory Picture’ of an environment. What also seems to matter is which ‘Type of Aid’ a client could benefit from, the client’s level of ‘Confidence’, the ‘Instructor–client Relationship’.

#### 3.1.1. Systematic Planning

Something that virtually all O&M instructors talked about is a combination of processes here dubbed ‘systematic planning’. These processes include noticing a certain sensory cue, then interpreting this cue and acting upon this judgement, as well as anticipating the cue that needs to be encountered next. Improving this systematic planning skill can increase a client’s confidence when they realise that they are able to trust their non-visual senses, as well as solidify the client’s sense of orientation (i.e., knowing where one is, and what one is perceiving). This systematic approach to route-following is best summarised by the following passage:

*“Doing certain actions in a certain order. […] ‘If I keep following this wall, then I have to- when I arrive at this foliage, I know: after the foliage, I turn right. And then I have to first follow the bend in the road and if I then feel this electricity box, I turn left, because then I can cross the street without ending up off-road’.”—Instructor 10*

*Noticing cues*. O&M instructors explained that some clients are better than others when it comes to noticing certain cues in the first place (see also ‘Non-visual Sensory Skills’ sub-theme). Improving one’s cue noticing skills is important for orientation (e.g., using objects to orient oneself), mobility (e.g., being able to notice the cane rolling down a slope to anticipate this slope), safety (e.g., stopping when the cane moves across some blistering tactile paving instead of continuing to walk), and flexibility (e.g., being able to recognise a cue that tells them they have missed an earlier reference point). Attentiveness to the environment is key for this step according to the instructors, as they find that the chance of recognising the next landmark increases when the navigator has a high level of attentiveness to their surroundings. This relates to the ‘Mental Capacities’ sub-theme, in the sense that it varies per person how much concentration they are able to muster. In some cases, a client is not attentive enough. In these cases, a person is more likely to become distracted by irrelevant cues or trains of thought, or by the urge to make small talk with their instructor. In other cases, a client is too concentrated. This can result in a person only focusing on the here-and-now of the route instead of focusing on what is happening in the general environment to generate a spatial overview. Instructors emphasised that an individual’s mental state appears to be a prerequisite for effective non-visual sensory information during navigation, as it might influence which types of cues are more likely to capture the client’s attention. As instructor 03 elaborates:

*“She has difficulties with the emotional weight of using a cane, very understandable, heavy, and that impacts the ability of properly using her sense of touch. So that also adds to it. Yeah, so sometimes it is not necessarily whether or not she can use these senses, but also if she is emotionally able to surrender to that.”—Instructor 03*

Interpreting cues. Many clients that O&M instructors work with have little to no experience with using non-visual cues for navigation. These clients have to learn to interpret these non-visual cues, as they would benefit from knowing which sensation belongs to which type of material or object. Instructor 01 described how the interpretation of cues can work in practice:

*“How would you label that? And if then- some indeed say, like, ‘yes, I think it is a tree’, you say, like, ‘okay, then call it- yes it is a tree, call it ‘tree’ and next time try- so the moment you get this same feeling again, try to check for yourself, like, is it indeed a tree I’m feeling and with that you actually complete your picture, you’re going to recognise feelings or sounds or things’.”—Instructor 01*

Acting upon cues. Knowing what you are perceiving makes it possible for you to act upon this knowledge appropriately. Examples from the instructors’ stories include being able to build in checks for maintaining one’s route, determining one’s orientation within an environment, knowing when to take a turn, and—if an object is labelled as an obstacle—to avoid an obstacle. Some clients can do this fairly automatically, though some participants do not take the appropriate action even when they do notice and properly interpret a navigation-relevant cue. For example, when feeling an unevenness in the walking surface with their cane, they keep on walking in the same direction instead of making the more appropriate decision to walk around it. Participant 03 commented on individual differences in how clients act upon cues they encounter:

*“Some people really need to walk slowly to let that information sink in, so they can go around the bollard. But there are also people who only need a little tap on the bollard and they’re immediately on their way. And then I don’t want them to slow down at all, because, well, they’ve interpreted it correctly and they can, they can continue.”—Instructor 03*

Anticipating cues. After noticing, interpreting, and acting upon a certain route-relevant sensory cue, the next landmark should be sought out. Indeed, an important part of the systematic approach to route-following is that a step-by-step plan needs to be held active in working memory (see also ‘Mental Capacities’ sub-theme). So, a client needs to learn which consecutive cues to look out for, and which consecutive actions to take in order to arrive at their destination. Systematic route-following therefore involves constant conscious monitoring of one’s current location (spatial updating) during prolonged navigation. As with noticing, interpreting, and acting upon cues, instructors also observe individual differences in skill regarding the anticipation of relevant cues:

*“If I then notice, like, ‘well, this person was not necessarily searching for that reference point, then I also try to practice, like, right, the moment you’ve passed one reference point, already start thinking, like, ‘okay, what do I need to expect in a bit to know where I am?’ Yeah.”—Instructor 07*

#### 3.1.2. Completing the Sensory Picture

Besides the constant monitoring of the environment for specific cues, a complementary component of O&M instructor’s training approach is teaching clients to ‘complete the sensory picture’. This entails the active construction of a representation of either a situated cue, a specific location, the spatial lay-out of an environment, or the route as a whole through the combination of information from multiple senses. The extent to which O&M instructors emphasise haptic or auditory information and how they integrate input from multiple senses varies across individuals. However, most instructors did emphasise that all sensory modalities ultimately contribute to forming a coherent understanding of the surrounding environment. One strategy that instructors use to teach clients to ‘complete the picture’ of a route involves actively implementing checks along the route. These checks involve verifying one’s position through multiple senses, for example, confirming the presence of a landmark or location, particularly in areas that are difficult for a specific individual. Regularly applying such checks reduces the likelihood of missing landmarks or getting lost and increases orientation (i.e., knowing where you are). In turn, this heightened awareness of where you are along a route fosters a greater sense of security and confidence, and enables more informed and goal-directed wayfinding decisions as long as the systematic approach is followed. The following piece of example data illustrates how the ‘completing the sensory picture’ strategy can work in practice:

*“You cannot rely solely on your ears, you cannot solely rely on your vision, you cannot trust your smell, you cannot trust the sun or the wind, but it does all complement each other. And that’s what it’s like. From the moment you feel something with your cane and you see a shadow of a tree in your field of vision and you see that the light isn’t there and the wind is diminished… Then the complete picture can be: ‘hey, I arrived at the tree’.”—Instructor 01*

However, we also found that multiple instructors primarily focus on teaching clients how to use their sense of touch for navigation. One instructor, for instance, gave the reason that recognising the environment via cane use is sufficient for route following. Another instructor explained that shorelines are trustworthy, whereas other non-haptic sensory cues are often too unreliable for navigation (e.g., the smell of a bakery, or the direction of the sun). In other words, only haptic cues would then be able to serve as anchor points (i.e., stable points of reference within an environment). According to these instructors, other sensory modalities (especially sense of hearing) come second, and are useful inasmuch as they can provide additional information when needed. That is, combining information from multiple senses could be useful in case of potential danger, such as when crossing a street. Some instructors indicated that they also explicitly teach clients to use multiple senses when a route becomes difficult, for example, one instructor who explained their instructional strategy when the client does not seem to be able to independently follow their route:

*“It’s often when I notice a route is getting more difficult, so if someone hasn’t yet… The route hasn’t ‘clicked’ for them yet. […] They say: I’m thinking to the left here. And then I say: okay, how can you recognize that? [laughs]. What else are you looking for, or what are your landmarks to… What are you looking for? Or what are you feeling right now? […] Then you really start to combine those senses or add extra points to, well, make the route more complete.”—Instructor 08*

Finally, one instructor added the nuance that this ‘completing the sensory picture’ strategy not only depends on individual differences in how well a person is able to pick up certain sensory cues, but also on the characteristics in the environment. Indeed, in quiet areas (e.g., rural areas) there are fewer sounds to even consider using for wayfinding. In contrast, busy areas (e.g., market squares) might only have useful auditory cues while lacking any useful haptic cues. In this way, the combination of personal and environmental characteristics can inform the instructor’s route choice to their client’s desired destination.

#### 3.1.3. Type of O&M Aid

Besides cane use and guide dogs, participants discussed smartphone applications, tactile maps, and audio recordings. Though additional wayfinding technologies might benefit some clients, these technologies are often focused on orientation rather than mobility aspects. Participant 05 in particular, who was especially interested in mobility aspects (e.g., teaching clients to identify walking surface irregularities to prevent falling), saw mostly disadvantages of using wayfinding technologies besides the cane and guide dog. Besides the lack of mobility-focused technologies, this participant also mentioned that wayfinding technologies are usually too expensive to use, that the technologies rarely enter the consumer market, that GPS signals are often not accurate enough for visually impaired individuals, and that signals resulting from these technologies might be distracting or annoying, as it is yet another cue that the navigator needs to pay attention to. In terms of smartphone applications, the participant mentioned that the route chosen by a certain navigation app is not necessarily the best route for the client. However, they added the nuance that some clients like using these apps, as it provides a guarantee that they will arrive at their destination eventually, even though the route towards it is unknown.

Cane use. One of the major pillars of O&M training is teaching clients how to benefit from a white cane, and learning proper cane techniques. Whether a client is ready to step outside with a cane can vary greatly between clients. Instructors explained that this is largely influenced by emotional aspects, such as loss processing and social implications. In terms of loss processing, transitioning to cane use can make the impairment feel ‘more real’. Having the courage to use the cane also means having the courage to admit that you need the cane to be able to follow your routes instead of relying on vision. Possible benefits of relying on the cane rather than residual vision are that it saves energy and brings a sense of security by confirming suspicions and landmarks. Some clients might not be fully ready to use the cane at the start of training, but express relief and joy upon realising how much freedom their cane can provide. Though, if it turns out that a client is not sufficiently ready yet for a cane, psychological help might be advised during or before continuing with O&M training. In terms of social implications, transitioning to cane use makes the impairment (more) visible to others. In practice, this means that other people will react to clients, both in positive and negative ways. Instructors notice that some clients are afraid of negative reactions from others, but during training experience that some feared responses do not occur or even experience positive interactions, which strengthens their courage to keep using the cane. For example, a client might fear people’s reactions when accidentally hitting them with their cane. Still, clients do encounter unpleasant or even dangerous situations due to the behaviour of other traffic users. Whereas most traffic users make space for a cane user and car drivers usually stop for a cane user that wants to cross a street, it does occur that clients collide with other traffic users who are focusing too much on their telephone screen while walking and subsequently react negatively towards the cane user, or that car drivers do not stop or even accelerate when a cane user is trying to cross a street. So, even though cane use might ease a visually impaired person’s wayfinding process, there can remain some legitimate fears surrounding cane use in public that can pose considerable psychological hurdles for actual cane use.

We found that when a client demonstrates readiness to use a cane, or is already doing so when starting O&M training, both their level of experience and the type of cane employed are important considerations. The specific cane design determines the nature and extent of information available to the user. For instance, a ball-tip cane primarily conveys haptic information by allowing users to detect variations in walking surface texture, while the rolling motion simultaneously produces distinctive auditory cues. This design also facilitates the detection of surface irregularities such as loose tiles or holes. By contrast, a tapping cane involves a large sound-based component. It enables users to identify obstacles through the auditory feedback generated by tapping against objects or through echoes resulting from nearby objects when tapping the cane on the walking surface. Beyond these functional differences, canes also vary in comfort-related properties, including weight, sturdiness, and handle material. Furthermore, individual users differ in their perceived ease of use and preference for a ball tip versus a tapping cane.

Guide dog. According to the instructors, a guide dog and a cane serve fundamentally different functions. The cane acts as an obstacle detector, while the dog acts as an obstacle avoider. Whereas cane use involves navigating from one reference point to another primarily through haptic feedback, walking with a guide dog often entails moving more freely through open spaces, guided mainly by memory and auditory cues. With a cane, a person is constantly searching for the next obstacle or reference point. In contrast, a guide dog might do the obstacle detecting and reference point searching in the owner’s stead, meaning that a dog can potentially save a lot of energy, fasten the walking pace, and bring a sense of freedom to the owner. In terms of saving energy, the reduced cognitive effort associated with guide dog travel might make it more appealing for clients to rely on their residual vision, which would normally take up too much energy. Regarding walking pace, a faster pace requires advanced O&M skills and sufficient mental space for noticing and interpreting sensory cues during route following. In other words, the navigator should be able to make the same navigational decisions as with a cane but with less sensory information and in a shorter amount of time. Finally, the perceived sense of freedom afforded by a guide dog comes with a trade-off. Namely, clients must relinquish some degree of control and surrender to the dog’s guidance, which is easier for some clients than others.

Most importantly, instructors agree that before learning to walk with a guide dog, the client first has to learn the route by heart themselves and have a proper cognitive map in place. Indeed, a client can only give commands to their dog if they themselves memorised the route so well that they know which commands to give at which points along the route. So, the owner often needs to associate the reference point of their dog to an action along the route (e.g., knowing when to give the command ‘search bollard’, and subsequently know that you have to cross the street when arriving at that bollard). To this end, the instructor can first teach the client how one would walk a certain route with a guide dog using a Dog-Sim, a dog-sized cart on wheels that is being held by the client but steered by the instructor. This procedure is followed even when the client already has a dog, for the reason that making mistakes during training could cause confusion for the dog because they might memorise the wrong turn as the correct route. As a consequence, guide dogs are most beneficial in familiar environments, where the dog owner is able to give clear commands to the guide dog.

*“They think: ‘oh, handy!’. But the dog doesn’t determine- the dog does, does not take anything of your plate, except for stopping at certain locations and avoiding obstacles that were previously annoying, but did indicate where you were. So you miss obstacles that indicated where you were. And that dog does not indicate where you are.”—Instructor 10*

Furthermore, social stigma also plays into the type of aid a person might choose. That is, there is often an association of feeling pity for someone when they are seen navigating with a cane, but it is seen as something beautiful when collaborating with a dog for navigation. For people who are sensitive to other people’s perceptions or reactions, this might add to their desire to obtain a guide dog.

Last, the interplay between the personality of the dog and the client’s personality matters. Pairing a relatively stubborn dog with a relatively hesitant person might lead to some undesirable wayfinding outcomes, such as travelling in unwanted directions or delays due to the dog being distracted or even unwilling to move.

All in all, not every client would benefit equally from navigating with a guide dog. Individual differences regarding lifestyle and tendency to depend on residual vision, prioritising a sense of freedom, efficiency, and speed, the need for control, the ability to construct cognitive maps, sensitivity to social stigma, and personality all play into the choice for obtaining a guide dog or not (see also ‘Personal Contextual Characteristics’ theme). Instructors first teach a client how to walk independently with a cane, and afterwards assess the possible benefits of a guide dog if the desire for a dog is expressed by the client.

Smartphone apps. Smartphone applications can serve as valuable supplementary navigation tools for certain clients with visual impairments. Instructors noted that such tools are particularly useful for individuals who are already proficient cane users, who use a guide dog, or who possess strong technological skills. For instance, some clients are already able to use a smartphone for other purposes, such as working with text-to-speech or enlargement software. Clients with a well-developed cognitive map of their environment may also benefit, as they can match digital information of a spatial lay-out to their existing spatial knowledge. Instructors observe that smartphone-based navigation tends to be adopted more often by younger clients, typically under the age of 60, and by those who wish to travel independently across longer distances, such as between cities (see also ‘Priorities and Lifestyle’ sub-theme). Two instructors brought up the N-Vibe specifically, which is an app connected via Bluetooth to a device worn around the wrist that tells a participant which direction they need to go via vibrations on the skin. One instructor focused on the possible benefit of such a device. Namely, it frees up cognitive space to use sound cues from the environment, in contrast to navigation apps that rely on auditory-verbal instructions. The other instructor, however, also focused on the possible drawbacks of devices or apps in general. Namely, even though such technologies can expand someone’s world, it does give additional information on top of the already relevant environmental cues, so they make a bigger appeal to the existing skills of the navigator. Therefore, it usually takes quite some time to get used to new technologies, which can make navigation even more complex during this habituation phase.

Audio recordings. When ‘Systematic Planning’ is halting and someone is not able to use a smartphone, it might be a good solution for the instructor to record auditory-verbal route instructions for a client. That is, some clients do not have the mental capacity to remember which action to take when encountering relevant cues along their route. In that case, instructions such as ‘follow the wall until you encounter this and that landmark’, and when the client has reached the landmark ‘turn left at this and that landmark’, might help them get to their destination without overloading their cognitive capacity. Another situation in which this method might be employed is when a client has to walk important yet rarely travelled routes (e.g., going to the dentist twice a year). Last, clients who are highly motivated, are capable of using a recording device, and have a relatively high need for independence can also record their own routes for themselves.

Tactile maps. Instructors can choose to incorporate tactile maps into the training. Tactile maps can be useful when a client lacks the physical stamina to complete an entire training session, when clients wish to practice independently between training sessions, as a safety measure for situations in which they cannot determine the next step in a route, or to support the development of a cognitive map of the broader environment. One instructor, for example, described working with a client who had grown up in India and therefore had to learn from the ground up how streets in the Netherlands connect, what typically lies between them, and how local residential areas are spatially organised. In this case, a tactile map provided valuable additional information for the client to be able to orient themselves within the neighbourhood.

#### 3.1.4. Building Confidence

O&M instructors adapt their training strategies to clients’ differing levels of self-confidence, balancing encouragement with caution. They emphasised that the expression of confidence varies widely across individuals. Some clients, for instance, display excessive confidence or impatience, driven by a strong motivation to regain independence and explore new routes. In such cases, instructors aim to temper this eagerness by first consolidating foundational mobility skills, such as proper cane techniques, before expanding the scope of travel. In contrast, clients who exhibit fear or reluctance to leave the home might require gradual exposure and reassurance to build trust in their own abilities and sensory feedback. Some instructors also described the strategic use of positive reinforcement when a client has low self-confidence, noting that particularly younger clients may respond well to explicit rewards.

We found that O&M instructors adapt their training approach according to their client’s perceived level of self-confidence: they decide per client when to let the client discover the route or their erroneous ways themselves by trial-and-error, or when to intervene. Clients that overestimate themselves may benefit from encountering obstacles that reveal their limits. In contrast, more hesitant clients may gain confidence through small, successful experiences, because it can be very discouraging for people with low self-confidence to constantly make mistakes. In these cases, an instructor would gently intervene by asking questions or providing guidance when they anticipate a mistake, and refrain from intervening when they anticipate success. Learning by success will benefit these clients’ levels of self-confidence more, whereas others would benefit more from discovery learning (i.e., encountering problems and solving them independently). One of instructor 09’s experiences exemplifies how impactful self-discovery learning can be, and how it ties into an instructor’s own perception of training success:

*“Then she said: ‘I still want to thank you, because, yeah, you really let me discover for myself’. […] Instead of ‘everything just happens to me and I can’t do anything myself anymore’, she’s like ‘I make conscious decisions and I am capable of learning everything. I can still do it’. So for her further process, I thought: ‘yeah, those two mobility training sessions are not a lost cause at all’. It’s just that you did something wholly different than teaching her the route.”—Instructor 09*

When intervening in case of a mistake, instructors also reported adapting their communication style according to each client’s confidence level. For instance, instructors report communicating in a direct manner with clients who are already more secure or motivated to navigate independently, such as directly correcting errors (e.g., “you missed your landmark”). By contrast, instructors often asked less confident clients reflective questions instead, such as “where do you think you are right now?”, to encourage independent reasoning and reduce anxiety. Clients who demonstrated lower self-confidence, often younger individuals, were said to benefit from verbally discussing the systematics of the route (i.e., the step-by-step verbalisation of route elements, such as explicitly naming relevant sensory cues):

*“Sometimes, I also just let it go wrong. Or I say: ‘well, I have half an hour, start searching!’. Yeah, and that might sound crude sometimes, but yeah. And if someone then really- a young person of around 19 years old, also the one with those self-confidence issues, let’s say… Well, then I also really ask: ‘what do you notice?’ And then I try to really verbally check like, ‘hey, what are you feeling right now? And what should you be feeling? What are you looking for?’—Instructor 08*

#### 3.1.5. Instructor–Client Relationship

The quality of the interpersonal connection between an O&M instructor and a client, i.e., the extent to which they ‘click’, influences the perceived effectiveness of the training process. Establishing trust between the client and the instructor is viewed as a foundational aspect of this instructor–client relationship. One instructor in particular found it incredibly important to start establishing trust as soon as possible. In this instructor’s experience, gaining the trust of a visually impaired person might take one to two years, and it can never be restored once lost. Instructors noted that a strong rapport can facilitate faster route acquisition, reducing the need for repetition and additional training sessions. Conversely, the absence of such a connection may make it more difficult for instructors to attune to a client’s learning style and to identify what strategies would be most effective. As one participant reported that some clients need only three sessions, whereas others might need fifteen, and attributed this difference to the instructor–client relationship:

*“[thinks]. Yes, there’s a bit of that personal connection sometimes, I think, that’s what it’s about. I think that differs- I think that one client, for example, learns more easily with a colleague, with my other colleague, than with me. I think there’s a real difference in ‘how far you can get’ with someone, so to speak.”—Instructor 02*

Furthermore, an individual client’s priorities as well as preferred dynamic can shape how an instructor feels and responds during training. For example, clients that are highly motivated to navigate independently might react negatively to interventions of their instructor. Mismatches in preferred interpersonal approach may at times create tension or impact the instructor, particularly in social situations where onlookers’ reactions are perceived as judgmental:

*“Then I want to intervene, for example where there are all these bikes at a store. And, and then one time he, well angry is not the word, but he said like, ‘what will I do tomorrow then, when you are not around?’. Then I thought, ‘yeah, you’re right about that’. Although I do notice that passersby in the city look at you because he bumps into a bicycle, like ‘why aren’t you intervening?’.”—Instructor 04*

We found that O&M instructors differ in their default approaches to training due to a different perspective on the role of an instructor in an instructor–client relationship. Based on our analysis, we distinguish between a ‘collaborator’ role and a ‘teacher’ role. In the former, instructors view the instructor–client relationship in the basis as a partnership, in which the O&M instructor’s job is to add to the client’s own problem-solving strategies, rather than a fundamentally hierarchical relationship in which the instructor often ‘knows best’ and replaces the client’s existing strategies with the ones they find most suitable. These roles also relate to different attitudes towards unteaching (i.e., the process of helping the client unlearn previously acquired knowledge, habits, or behaviours that are incorrect, unhelpful, or no longer relevant). One instructor who seems to lean towards the collaborative approach says that they never deliberately unteach strategies that clients have taught themselves, they only add to them. The only exception is when a self-taught strategy causes any chance of danger. Another instructor unteaches something when it drains energy (e.g., relying too much on residual vision), or in potentially dangerous situations like taking chances when crossing the street (e.g., knowing for sure—rather than assuming—that a car has stopped). Yet another instructor discussed unteaching counting steps as a navigational strategy, and replacing it with the systematic planning strategy (i.e., noticing, interpreting, acting upon, and anticipating sensory cues in the environment; see ‘Systematic Planning’). Last, an instructor mentioned planning to unteach something at the end of a training trajectory, namely a client asking for too much confirmation instead of trusting their own non-visual senses (see ‘Building Confidence’, ‘Personality Traits’, and ‘Non-visual Sensory Skills’ sub-themes for more findings concerning the influence of a client’s level of confidence and trust on training strategies). The collaborative training approach is best elucidated by this excerpt from instructor 07’s interview:

*“I like it best when it really feels like you’re collaborating with the client, so you’re not just the professional, but the client is the one figuring things out, and I’m a complement to that. So, I think that’s a really nice idea, if that’s how it goes.”—Instructor 07*

Regardless of an instructor’s default approach, every instructor prioritises their client’s physical and emotional safety. Still, instructors vary amongst each other with regards to their risk assessment and their tendency to take on a protective, or ‘rescuer’, attitude. We observed that, consequently, instructors vary regarding how much autonomy they allow clients during potentially risky situations, such as crossing a street. Some instructors described their own anxiety and doubts about whether they dared let the client navigate independently or whether they should intervene to secure their client’s safety. O&M instructors were also conscious about ensuring training did not have any adverse effects on the client’s mental health (e.g., a decreases in self-confidence due to a lack of success experiences, or even causing any trauma or anxiety).

Together, these observations illustrate that O&M training is not merely a technical process of skill acquisition, but an inherently relational one. The social context of O&M training involves interpersonal dynamics that are unique for each instructor–client relationship. These dynamics are experienced as impacting instructional strategy choice, the duration of training, and perceived training outcomes.

### 3.2. Personal Contextual Characteristics

The second main theme describes personal characteristics of the client. Within this main theme, O&M instructors mentioned the relevance of varying ‘Ages’, ‘Priorities and Lifestyle’, and ‘Personality Traits’ of their clients. We found that these characteristics are relevant for O&M behaviours in the sense that they are indicators for the instructors regarding what a client wants to learn or is willing to learn. Namely, some clients live more active lives and navigate more often than others, sometimes due to anxiety, but other times due to age (either related to social or societal expectations, the motivation to learn, the type of locations that they wish to reach, physical limitations, or speed of learning).

#### 3.2.1. Age

Age came up as an influential demographic in terms of O&M. O&M instructors notice that older clients tend to have more limits in terms of physical conditions (see also ‘Physical Capacities’ sub-theme), mental conditions (e.g., memory and overstimulation; Mental capacities sub-theme), and status of their remaining sensory modalities (e.g., diminished hearing of high-pitched notes, such as bicycle bells in traffic; ‘Non-visual Sensory Skills’ sub-theme). As a result, O&M instructors might prioritise shorter rather than longer routes for older clients.

Furthermore, instructors correlate age with the ability to construct cognitive maps. Older clients seem to have better map-making skills compared to younger clients. A possible reason for this that participants came up with is that older clients have learned to memorise street names and lay-outs when they were young, as opposed to youths in the current day and age, who are often used to following the step-by-step instructions from smartphone applications without an urgent need to memorise spatial lay-outs.

Finally, age is closely connected to the sub-theme ‘Priorities and Lifestyle’. O&M instructors noticed that older clients tend to be more averse of travelling by public transport or using a smartphone as an O&M aid compared to younger clients. Therefore, older clients refrain from travelling to faraway or unfamiliar places more often than younger clients. Furthermore, O&M instructors find differences in social network that impact daily mobility depending on age, such as which people in their social network are able to help the client get outside and practice their route in-between training sessions. Moreover, older clients tend to have less societal expectations placed upon them compared to younger clients, such as being expected to learn how to independently go to the supermarket. If a client’s children are part of their social network, the age of the children also plays a role. Young children who are walking along will present the client with an added responsibility during route-following, whereas older children might be able to support their visually impaired parent instead. Though, older clients tend to be underestimated by the people close to them, such as their adult children, in terms of what they are expected to still be able to do independently or not. These expectations could influence the clients’ wants and needs concerning the destinations they wish to reach:

*“Well, people say like: ‘yeah, I don’t have that long [to live] anyway. So why should I invest in that?’. While if you are 35, or 40- Yeah, who is going to idle away the years when they are 40, 50 years old? They exist, but that, that, that societal expectation is different then.”—Instructor 10*

#### 3.2.2. Priorities and Lifestyle

O&M instructors point out individual differences regarding clients’ priorities and lifestyles that impact their O&M behaviours. Some people are more adventurous and might have more ambitious navigational goals, e.g., wanting to traverse the entire country instead of solely learning the route to the neighbour’s house. Clients who live more active lives often appear to be more motivated to learn the routes and practice in-between training sessions, and often express a stronger desire for independence compared to clients who do not engage in many activities on a daily or weekly basis. However, some lifestyle choices are not a matter of desire, but a matter of necessity. Some clients navigate on a near-daily basis due to their responsibilities. One instructor gave the example of a man who became visually impaired fairly suddenly, and taught himself a non-optimal navigation strategy out of necessity:

*“Yes, that sir has become visually impaired very suddenly and he is an informal caregiver for his whole family. So everyone there has a disability, only he didn’t have one until then. And, and he has to, yeah, he has to do the groceries, so he rapidly started to think, like, ‘how do I get to that store? Safely?’. So he figured that out himself, how many steps to take. Before we started walking with the cane. So he, he still does that.”—Instructor 05*

Lifestyle also influences which type of navigation aid an instructor would recommend to a client., e.g., the instructor will assess whether the client already was an avid recreational walker when a client says a guide dog would help them go outside more to maintain their routes, because guide dogs need sufficient physical activity and at least one and a half hours of work activity every day. And one instructor mentioned that you also need to be fond of dogs: after the work is done, the dog is also just a pet. Furthermore, instructors observe that someone’s lifestyle does not necessarily correlate with someone’s visual functioning or level of anxiety concerning independent navigation:

*“I do have access to a client’s visual data from my research, but I always try to also ask someone: how does that work in practice? Because, yeah, one person participates in running competitions with only 2 percent [residual vision], and another person doesn’t dare to take one step out of their house with 10 percent.”—Instructor 10*

Last, we found that some clients might be more in favour of using sensory cues for efficiency, whereas others focus more on phenomenologically enjoyable experiences. This focus can match or mismatch the approach of a particular instructor. That is, some instructors explicitly focus on the enjoyableness of some sensory information, regardless of its usefulness for navigation. Clients who prioritise efficiency might react negatively to this kind of approach. Alternatively, a cue pointed out by an instructor due to enjoyableness can become a useful landmark for navigation, against the instructor’s expectation. Other instructors predominantly focus on the necessary information for the route being currently taught, for example, because they do not want to overload a client with seemingly redundant information. Still other instructors like to ‘expand their client’s world’ beyond merely the route to one destination. They point out extra information when the chance exists that the client might benefit from that knowledge in the future, such as noting at some point along the route that there is a hairdresser across the street. Clients who might react well to this approach are clients who are exceptionally curious or who have a need for a lot of information as they are reported to already ask many questions about the spatial lay-out or sensory cues they encounter. One instructor elaborated on how this preference for enjoyable experiences and sensory information might manifest during training, and how it shapes his training approach (see also Building confidence and Instructor–client relationship):

*“I often let blind people touch the magnolia tree, for example, with its beautiful, huge blossoms. But they don’t see them, I’m assuming they’ve also never held them. So, I try to see if they’d like to touch them sometime. So it’s not just mobility I focus on, you can also alternate it with other things that are fun and a bit of a break, that build confidence and create a cosy atmosphere. That’s important too. Having a cup of coffee every now and then. That’s also possible, and important too.”—Instructor 06*

#### 3.2.3. Personality Traits

In terms of personality traits, O&M instructors often brought up how anxious, assertive, and flexible a person is.

Anxiety. Anxiety, both state and trait, is a broadly covered theme amongst the interviewed O&M instructors. They associate clients’ anxiety levels with their attitude towards mishaps, such as taking a wrong turn, missing a landmark, or encountering unexpected construction work. On the one hand, they observe that some clients have a better ability to stay calm in the face of adversity than others, and on the other hand, that some clients are more likely to search for solutions instead of asking the instructor for help in unexpected situations. One O&M instructor told a story about a client that panicked when crossing some train tracks when the train track’s alarm bells started to go off. The client froze while standing on the tracks with the train approaching, while the only thing the client should have done was keep on walking. Acceptance, perseverance, giving up, panic, and disappointment are reactions to mishaps that instructors talked about. Strategies instructors employ to decrease wayfinding anxiety could be to teach clients to trust that they are capable of solving problems, and to push them a little further than what the client themselves thinks they are capable of by reminding them of the activities and hobbies they used to have and encouraging them to try and pick these up again.

Furthermore, anxiety relates to self-confidence, in the sense that higher levels of self-confidence are associated by the instructors with lower levels of anxiety. Specifically anxiety shows in body language such as posture and pace, as people with higher anxiety levels tend to take up less space and move at a slower paces. Changes in body posture and pace are how many instructors indicate they see progress in their clients., e.g., clients will walk with a straight posture instead of hunching down staring at the walking surface, and pick up some speed when they are starting to feel more confident (see also ‘Building Confidence’ sub-theme). Besides body language and pace, another telling cue of self-confidence is how often a client would ask for confirmation or feedback during route-following. Though, O&M instructors also emphasise that how anxious someone is, or how little confidence someone has in their own capabilities, does not always reflect their actual capabilities. Clients are experienced as both overestimating their own O&M capabilities, for example, because they are not ready to admit they cannot perform the same tasks they used to, and underestimating their capabilities, for example, due to a lack of insight in how much they already make use of their non-visual senses. Last, O&M instructors seem to relate a client’s level of anxiety and self-confidence to the amount of mental space and focus necessary for spatial awareness (see also ‘Completing the Sensory Picture’ sub-theme):

*“What I do notice is that the client with the most peace and confidence has the most possibilities. The one who is a bit more stressed, he is only occupied with the cane and, and with the route, but not with the rest. So then that I- that they are like, ‘yeah, I have no idea where I am’. And that I really have to start asking questions, like: ‘okay, what, what do you hear then?’.”—Instructor 01*

Finally, clients’ levels of anxiety are observed by instructors to strongly influence their attitudes toward independent navigation in daily life. While some individuals experience such high levels of anxiety that they rarely leave their homes, others might even venture out along routes they have not formally learned to navigate using their non-visual senses, often relying on (visual) memory or estimation instead. They are confident that they would be able to properly solve any problems they might encounter. On the one hand, this exploratory approach could be beneficial for a client’s navigation skills, as instructors emphasise that it is a skill that requires continuous practice and avoiding outdoor mobility may lead to a gradual decline in navigation ability. On the other hand, this exploratory approach often involves excessive risk-taking without adequate preparation, meaning that it increases the likelihood of disorientation, error, and dangerous accidents. Consequently, the degree to which a client achieves functional independence depends, in part, on their ability to balance initiative and caution when engaging in mobility activities. Instructor 02 explains:

*“It really helps when people only with us- if we have, let’s say, one appointment a week and they practice with us, but they don’t after that. Well, then the training takes forever. If someone has the courage or the thing like ‘I’m going to practice myself too’, things like that, then it goes much easier, much faster.”—Instructor 02*

Assertiveness. Clients vary in how timid or assertive they are. On the one hand, instructors indicate that young clients can lack self-confidence, be anxious, and tend to not assert themselves sufficiently. A client’s level of assertiveness (or courage) has an effect on their body language, behaviour, and attitude. Regarding body language, multiple O&M instructors describe how timid people do not want to take up much space and make themselves small or keep close to the wall instead of the curb when trying to cross the street, impacting their visibility for other traffic users and therefore their safety. Similarly, if someone crawls into their shell, other traffic users are less likely to part for them. Especially in busy environments, exuding self-confidence might make navigation significantly easier for the client as other traffic users will notice them sooner. Though, the other extreme also occurs, where a client assumes other traffic users will stop for him instead of taking a moment to signal with his body language and cane technique that he intends to cross. Another example regarding assertive behaviour includes a client who contacted one of their neighbours to adjust something in their garden, so they could more easily access an important shoreline. Regarding attitude, clients vary in how comfortable they are with accidents such as unintentionally tapping someone with their cane. One instructor pointedly said that there are thinkers and doers. Doers might bump into obstacles more often, but they will eventually reach their goal. Thinkers, on the other hand, might overthink crossing a street, and therefore lack the assertiveness necessary to ensure their own safety. A consequence relevant for navigation would be that a client that is uncomfortable with asserting oneself might not hold out their cane far enough for other traffic users to see.

Flexibility. Clients vary in their attitude towards change. For example, some clients have taught themselves a certain route, or they still know a certain route from the past when they could still see, and might have trouble with taking different routes, detours, or use new landmarks. Other clients can be too inflexible with regard to changing their own opinions on what the best navigational decision is at a certain point along a route. Consequently, several instructors indicated that, in selecting routes for instruction, they consider what a specific client is likely to do in real-world situations when the instructor is no longer present rather than what the client is formally taught to do. Finally, O&M instructors find that older clients in particular tend to have more difficulties with change compared to younger clients. For older clients, instructors therefore often prioritise optimising travel along familiar routes rather than teaching new routes.

### 3.3. Sensory Modalities

The third main theme we formulated concerns the use of one’s senses during route following. According to the O&M instructors, clients vary greatly in terms of which sensory cues they perceive. Not only does the ‘Visual Impairment’ itself influence an individual client’s O&M behaviour, their ‘Non-visual Sensory Skills’ also matter. How the sensory information is or can be used therefore also varies per client (see also ‘Systematic Planning’ and ‘Completing the Sensory Picture’ sub-themes).

#### 3.3.1. Visual Impairment

Instructors have access to the visual function test reports of their clients that include measurements regarding a client’s visual acuity, field of view, and other visual functions. Although instructors notice that these characteristics of the impairment do impact clients’ O&M behaviours, most instructors emphasise that they deliberately avoid making assumptions based on these pre-given reports about what a client can or cannot still see in practice. For each client, they assess individual challenges and abilities when using residual vision for navigation. Despite these conscious efforts, assumptions do still persist. We found that this goes both ways: instructors can assume their client can see a certain obstacle when it turns out they cannot, but instructors also mention being surprised when a client does see something the instructor thought they could not.

Instructors reported that clients with residual vision still tend to rely on visual cues as their primary source of information. For some, this reliance is linked to difficulty accepting that their visual input is not always reliable or sufficient, while others seem to be more visually oriented by nature. Several instructors indicated that they intentionally include visual landmarks when designing routes for such clients. However, excessive dependence on residual vision can have undesirable consequences. Instructors observed that it may hinder clients from learning to trust their non-visual senses. In some cases, clients with residual vision failed to notice salient auditory, haptic, or olfactory cues because their attention remained visually focused, often seeking to confirm non-visual perceptions through sight. This overreliance on visual input also makes it more challenging to foster consistent multisensory engagement during training. One instructor noted that clients with greater residual vision often exhibit more doubt or hesitation during navigation, whereas those with minimal vision or blindness tend to demonstrate stronger trust in their non-visual senses. Moreover, visual cues that are effective during daytime may become unreliable at night or under other low-light conditions. Last, sustained visual effort can lead to physical discomfort, such as eye strain, neck pain, or headaches. For these reasons, instructors emphasised the importance of gradually reducing dependence on visual landmarks relative to non-visual landmarks.

Onset. O&M instructors talked about two main ways the onset of the impairment influences navigational behaviours. First, they distinguished early-onset versus late-onset impairments. That is, clients with early-onset impairments often have more experience already with relying on their non-visual senses. O&M instructors mentioned that they notice a substantial difference in this regard between clients with early-onset impairments compared to late-onset clients. The former are perceived as more skilled in non-visual cue use (specifically regarding the interpretation of non-visual cues within the Systematic Planning approach), whereas this transition from using vision to using other senses for navigation tends to be more difficult for clients with late-onset impairments. On the other hand, instructors remark that clients with late-onset impairments tend to have more elaborate cognitive maps based on their visual memories of an environment compared to clients with early-onset impairments, making it easier to employ these maps as an extra tool during wayfinding (see also ‘Spatial Understanding’ sub-theme). Second, O&M instructors see that progressive versus adventitious impairments impact their clients’ wayfinding behaviour in a meaningful way. For clients with progressive impairments, instructors tend to anticipate the decline in functional vision by purposefully selecting routes or landmarks that are also doable for people with less residual vision. Age of onset relative to the age at which O&M training starts might also relate to clients’ anxiety levels observed during training. That is, instructors observe that clients with late-onset impairments are generally more anxious, which they conclude from observations such as lower travel frequency, slower walking pace, and slower obstacle detection in clients with late-onset impairments (see also ‘Personality Traits’ sub-theme). Besides more anxiety, clients with late-onset impairments also seem to struggle with other emotionally charged changes. This not only pertains to processing emotions around their vision loss, but also to a loss of independence and the sudden necessity of having to ask for assistance from passersby, loved-ones, or staff. Within the context of O&M, instructors noted that when clients are emotionally overwhelmed, they seem to have reduced cognitive space to orient themselves and to process environmental cues effectively (see also ‘Systematic Planning’ and ‘Mental Capacities’ sub-themes).

Visual field. Instructors take their clients’ visual field loss into account when applicable. For example, individuals with peripheral visual field loss, such as tunnel vision, may still acquire a substantial amount of visual information but can experience difficulties in situations requiring a broader spatial overview, such as crossing a street. One instructor indicated that, in such cases, it might be most effective to cross without relying on vision altogether, admitting that this can be anxiety-provoking for clients. In contrast, clients can have relatively stable vision in the periphery of their visual field while lacking vision in the central visual field (e.g., in case of macular degeneration). In these cases, clients are said to have a relatively large amount of visual information to draw upon while wayfinding. Last, people with altitudinal field defects in the upper quadrants will encounter problems such as walking into hanging tree leaves, whereas people with altitudinal field defects in the lower quadrants will encounter more problems with tripping or managing sudden height differences, such as curbs. It is the latter group of clients who could benefit particularly from learning how to use a cane, as they can use the cane for managing these challenges and consequently shifting their gaze from the walking surface to their general surroundings. Another factor influencing navigation, as reported by instructors, is the location of landmarks relative to a client’s visual field. For instance, when approaching from a particular direction, a landmark may fall within the left visual field. If the client fails to notice it, they may turn around to search, at which point the landmark would now appear in the right visual field where it may not be detected. In other words, missing a landmark can have a disproportionately greater impact on navigation for clients with left/right visual field defects. In some cases, this might have far-reaching consequences, such as cessation of the O&M trajectory:

*“And then she walks back, still not realizing that there’s a curve in the sidewalk. She’s missing the bollard, too, because she can’t see anything on the left. She only sees something on the right. And if that bollard is on the left, she has a hard time seeing it, and then she basically loses her way. That’s also why we stopped. I can’t get, can’t get that into her head, and it has to be safe for her.”—Instructor 06*

Light perception. The descriptions of the instructors imply that clients who retain some light perception may experience both advantages and disadvantages in navigation. For example, reflective surfaces such as bicycle frames can produce glimmers that are still detectable, which might, for example, promote safety when crossing cycling paths. Additionally, the ability to perceive light–dark contrasts or brightly coloured landmarks may support orientation. However, instructors noted that these possible benefits highly depend on environmental conditions. Diffuse lighting, as occurs on cloudy days, may reduce the utility of residual vision, whereas strong contrasts under sunny conditions may facilitate navigation. At the same time, excessive sunlight or considerable transitions between light and dark (such as when moving from indoor to outdoor environments on sunny days) can be overwhelming for clients who are prone to sensory overstimulation or distraction. As light conditions might vary from day to day, or even from moment to moment, O&M instructors notice that a single client might navigate excellently under some conditions, but fail at the exact same route when these conditions change.

#### 3.3.2. Non-Visual Sensory Skills

O&M instructors report that they often need to make their clients aware that they can actually actively use non-visual senses for navigation. They also recognise individual differences between clients regarding their abilities to use their non-visual senses during navigation, which influences which reference points instructors choose for their client. Being able to distinguish landmarks is an essential skill during wayfinding. Indeed, as several instructors indicated, navigation while visually impaired becomes challenging when environmental cues sound or feel too similar. Within the *Systematic planning* approach, this would mean that the pipeline from noticing to interpreting to acting upon sensory cues is halting in the interpretation step. Individual differences in sensory skill were observed in abilities relating to touch. For instance, instructors described individual variation in how effectively clients interpret haptic feedback through the cane, such as recognising the distinctive texture of blistering tactile paving at pedestrian crossings before physically reaching it. Clients who lack this skill as well as experience reduced sensation in their feet due to conditions such as diabetes may struggle to detect variations in walking surface texture. In such cases, instructors might shift focus from haptic to auditory cues, emphasising instead the change in sound produced by the cane when moving across surfaces. Instructors further reported variation in auditory perceptual skills. Some clients demonstrate refined echolocation abilities, enabling them to identify spatial features through reflected sound, whereas others are less adept at distinguishing or interpreting auditory cues. Similarly, clients differ in their capacity to identify olfactory landmarks. Instructors also noted that variation exists within, not just between, sensory modalities. For example, a single client might effectively notice, interpret, and act upon a tile–asphalt border through haptic feedback yet struggle to perceive differences in walking surface texture. These individual differences also shape instructional strategies. For instance, instructors pay attention to what an individual client notices themselves. If someone notices that they are walking past a tall building, then instructors know that henceforth, other tall buildings along the route might also be chosen as points of reference. In other cases, when a client is not able to distinguish between the stable haptic cues within an environment, the instructor might actively place stable cues from another sensory modality within that environment:

*“He was having trouble finding his house, his driveway: it’s all a bit similar. I put two posts in his yard, those, yeah, arm-thick posts with white tubes around them, and then he has to stand between the two white tubes. That’s his entrance, so to speak. That, he can see. Still, anyway.”—Instructor 06*

### 3.4. Capacities and Limits

The final main theme describes individual differences in what clients can and cannot do in context of O&M. These ‘Capacities and Limits’ are represented by three sub-themes: ‘Spatial Understanding’, ‘Physical Capacities’, and ‘Mental Capacities’. These capacities and limitations guide O&M instructors in personalising the training process by determining what is feasible in terms of route selection, cue use, technological support, destinations, travel frequency, and overall training approach.

#### 3.4.1. Spatial Understanding

The Spatial understanding sub-theme encompasses instructor’s discussions on individual differences between clients regarding their ability to understand and memorise spatial information and regarding their sense of direction and their sense of distance. For instance, O&M instructors reported notable individual differences among clients in their ability to form cognitive maps of their surroundings. Instructors shared that clients with more accurate or detailed mental representations of the environment are seemingly better able to use their sense of distance for orientation and to navigate flexibly within an area. Such clients are thought to be capable of understanding spatial relations, such as the connections between streets or the relative positions of landmarks, which in turn facilitates more efficient route planning and spatial awareness. For instance, when a client knows how certain streets intersect and recognises environmental sounds such as traffic flow, they may infer that they have reached a particular intersection and use this point as a reference for subsequent navigational decisions (see also Systematic planning sub-theme).

Furthermore, someone’s level of spatial awareness is noticed by instructors through their sense of direction. For example, some clients have rather good path integration skills, where they will be able to point quite accurately to the location of their house (i.e., the starting point of their route) or point to the North. Though, instructors point out that it matter show familiar a client is with the environment, e.g., for how long someone has been familiar with the environment, whether they have recently moved to a new environment, or whether they still have visual memories of an environment. Prior visual experience within an environment was described by the instructors as having both potential benefits and drawbacks. Namely, visual memory of an environment might serve as a back-up source of information during route-following, it might facilitate visualisation of the route, and it might benefit the integration of auditory or haptic information within the existing spatial representation. In some cases, however, prior visual experience interferes with the ability to learn the route haptically. Finally, instructors mentioned clients whose spatial awareness was so limited that they struggled to find their way even within their own homes. Such cases were believed to be exceptional, though they were mentioned by multiple instructors.

#### 3.4.2. Physical Capacities

There are some physical limitations of clients that O&M instructors talked about. First, a client may have additional physical disabilities that negatively impact the ability to walk for prolonged periods of time. Second, the energy levels of clients vary substantially. In particular, a client’s energy level will influence how long the routes can be. Moreover, instructors might select a route with a park bench around the halfway point for a client with lower stamina to rest on. Third, some clients have better balance than others. This ability is particularly necessary when managing holes in the road or loose tiles. Furthermore, instructors report that people who are easily overstimulated also tend to lose their balance more easily. Last, if clients sway, instructors will tell them to place their feet further apart to create a more stable base. Physical condition is associated by the participants with age, as they observe that available amount of stamina and ability to keep one’s balance typically decline with age, and that additional physical and sensory disabilities also occur more frequently in older clients (see also *Age* sub-theme). Together, these findings illustrate the intrinsically embodied context in which navigation takes place, as an individual client’s bodily capacities and limitations guide instructor’s route and instructional choices.

#### 3.4.3. Mental Capacities

O&M instructors reported individual difference between clients in terms of mental capacities. Specifically, they notice variation (working) memory capacity and learning capabilities, as well as attentional processes related to a client’s resilience. Instructors perceive memory capacity and learning capabilities as influencing how many sessions someone needs to learn a route, how much repetition is needed, or how much can be achieved in a single training session. In practice, this means that if a client is able to learn multiple routes in a relatively short amount of time, they have more possibilities for independent navigation in their daily lives.

Resilience, in O&M context, was used to denote the amount of effort that is required to process certain types or certain amounts of sensory information at once. As such, resilience was linked to the ability to maintain concentration while navigating, as this takes more effort for some clients compared to others. Some instructors described clients who easily lose focus, whereas other clients are able to focus on multiple tasks at once (e.g., performing mental calculations while finding their way with their cane without the use of shorelines). Furthermore, O&M instructors reported that relying on residual vision can be exhausting for clients due to the required level of information processing effort, and that this counts especially for clients with a fair amount of residual vision whose visual perception is not reliable. A focus on residual vision then involves constant doubt and monitoring. Other clients were said to have a low threshold for experiencing sensory overload from both visual and auditory stimuli. In such cases, instructors noted that it can be calming for clients to ‘let the cane do the work’, relying instead on the consistent haptic feedback provided by the walking surface. Clients who are easily overstimulated might have more difficulty navigating busy areas, which is why instructors report selecting detours through less crowded areas, or to advise the client to travel the route during a quieter time of day. Instructors emphasised that for clients who are easily overstimulated, concentration and navigation skills may fluctuate even within a single route:

*“Especially with clients with acquired brain injury there are obviously those overstimulation issues. And that means someone might be a great cane user at the beginning of their route, but suddenly they’re not halfway through because fatigue sets in.”—Instructor 03*

Challenges concerning mental capacities may be amplified in individuals with intellectual disabilities. For instance, an instructor might assess whether the client is able to memorise an association between a landmark and an action (as is part of the ‘acting upon’ step within the ‘Systematic Planning’ approach). If not, the instructor might gauge if a client is able to follow point-by-point instructions from a smartphone application. If that also fails, an instructor might choose to record their own instructions for the client (see also ‘Type of O&M Aid’ sub-theme). One O&M instructor noted that, although verbal communication can be difficult in some cases, it is still possible to observe meaningful behavioural responses when introducing a cane to the client. For example, some individuals may wield the cane erratically, posing safety risks to themselves and others, while others may start “running around” feeling a sense of newfound freedom. Though it can sometimes be challenging for instructors to introduce a cane to a client with intellectual disabilities due to attitudes of family members or caregivers. Namely, they may not believe that a client will be able to use a cane effectively, though some clients do demonstrate noticeable improvements in navigation with the aid of a cane. For instance, a person who previously stood still for a long time when wanting to cross a hallway—misattributed to the client’s dementia—was actually listening too intently and stopped hesitating to cross the hallway when given a cane.

## 4. Discussion

People with visual impairments (VIPs) have the option to receive Orientation and Mobility (O&M) training in order to learn how to safely and independently follow routes to their everyday goal locations. In this study, we interviewed O&M instructors to identify which individual differences between their clients they take into account when teaching them how to independently travel these routes, with a particular interest in personal characteristics and sensory cue use, and how these differences can shape their instructional practices. Based on our thematic analysis, we created four main themes that describe these relevant individual differences: ‘Personal Contextual Characteristics’ (particularly ‘Age’, ‘Priorities and Lifestyle’, and certain ‘Personality Traits’), ‘Capacities and Limits’ (both ‘Physical and ‘Mental’, as well as concerning ‘Spatial Understanding’), ‘Sensory Modalities’ (both related to their ‘Visual Impairment’ and their ‘Non-visual Sensory Skills’), and ‘Training Approach’ (i.e., how wayfinding strategies such as ‘Systematic Planning’ and ‘Completing the Sensory Picture’ are taught and how the instructor goes about ‘Building Their Client’s Confidence’, as well as how the ‘Type of O&M Aid’ and ‘Instructor–client Relationship’ play into the training approach). From the theme descriptions, we infer that the issues described within these themes dynamically interact during O&M training (see Figure 2 for a visual representation of these hypothesised dynamics).

### 4.1. Trust as a Facilitating, Formative, and Intrinsically Valuable Factor in O&M Training Process

Trust emerged as a central construct across all four main themes identified in this study, functioning as the integrative element, or conceptual ‘glue’, that connects them. First, self-confidence (reflecting the Dutch notion of ‘zelfvertrouwen’, literal translation ‘self-trust’; main theme ‘Personal Contextual Characteristics’) captures the general belief that ‘I can do this’ while travelling with low vision or blindness. Second, trust in one’s non-visual senses (main theme ‘Sensory Modalities’) reflects confidence in the ability to effectively use all remaining senses for navigation. Third, interpersonal trust between instructor and client enables cooperative communication and safe exploration during navigation (main theme ‘Training Approach’). Finally, we conceptualise these three dimensions of trust as a prerequisite for the cognitive space required to focus on the navigation task (main theme ‘Capacities and Limits’).

We speculate that, together, these interrelated forms of trust have a foundational role in supporting both the emotional and cognitive dimensions of independent mobility. First, the narratives of our participants suggest that self-confidence (in this context defined as trust in one’s general navigational competence) is involved when deciding whether and how frequently to engage with mobility tasks. Frequency of travel and travel duration are both associated with quality of life [23,34]. This is possibly due in part to the relationship between reduced mobility and diminished social participation, an issue also reflected in our ‘Priorities and Lifestyle’ sub-theme. Therefore, lower self-confidence might negatively impact someone’s quality of life due to lower travel frequency and less social engagement. However, during navigation itself, low self-confidence might also have negative consequences. That is, our participants reported that low self-confidence in their clients manifests in body posture and movement, which can influence how other road users perceive and respond to them. In this way, low self-confidence might have serious consequences for the person’s physical safety. The magnitude of this effect is likely to vary across individual clients. Namely, prior research suggests that gender and age interact in shaping self-perceived navigational competence. Van der Ham et al. [35], in a large-scale study encompassing sighted participants across the lifespan, found minimal gender differences in actual navigation performance but did find differences in participant’s own performance judgments. Men tended to overestimate their performance, an effect that increased with age, whereas women generally underestimated their abilities (except among older women, who tended to overestimate their performance). If similar patterns exist within the visually impaired population, then women under the age of around 70 years old could have a higher chance of lower quality of life and higher risk to end up in dangerous situations due to lower self-confidence. In sum, O&M instructors view their clients’ self-confidence and its resulting behaviours as playing an important role in both the actual navigation process and the emotional aspects related to navigation, which seem to be intricately interlinked.

The second dimension of trust involves a client’s efficacy expectations about their capability to perform specific wayfinding tasks, which consequently affects their decision-making behaviours [36]. In the context of O&M training, clients’ self-efficacy is particularly relevant when it comes to using one’s non-impaired senses for spatial tasks. Namely, our data suggest that many clients with low vision need to update their efficacy expectations of their ability to use their non-visual senses and need to learn to trust the feedback from their non-visual senses in order to navigate effectively. As individuals are more likely to confidently engage with tasks they believe they can manage and tend to avoid situations they perceive as beyond their capabilities [36], clients’ efficacy expectations might also play a role in their engagement with O&M tasks. For example, their self-efficacy might impact the transition from visual to non-visual cue use and the willingness to leave their home between training sessions to practice, which could in turn influence the speed and quality of skill acquisition. Moreover, challenges and mistakes will inevitable occur when learning a new navigational strategy such as using non-visual cues. So, one’s trust in their ability to cope with these challenges and mistakes could also determine their subsequent navigational decisions. These varying attitudes towards mishaps and their subsequent navigational behaviours are reflected in the ‘Personality Traits’ sub-theme, as well as in findings from our previous study which describe these patterns from the perspectives of VIPs themselves [2]. These findings suggest that, in practice, clients are more likely to make deliberate navigational decisions when they trust their capabilities rather than succumb to panic when facing challenges and become disoriented.

The third dimension of trust that occurred within our data is that O&M instructors described the importance of establishing a relationship of trust with their clients. We attribute this finding to O&M instructor’s focus on physical and emotional safety. On the one hand, physical safety seems to be the instructor’s highest priority in general during route training with visually impaired clients. This focus on safety aligns with findings by Asave et al. [37] and Bandukda et al. [15], who reported that the O&M instructors in their studies tend to prioritise safety over autonomy (i.e., encouraging a client to ask for assistance rather than attempting to navigate independently) and over efficiency (i.e., encouraging a client to take a longer route if it is safer than the shorter route). It would make sense, therefore, that instructors would want to ensure their clients’ physical safety by ensuring that their clients trust their help and their judgments. On the other hand, a client’s trust in their instructor forms the foundation upon which VIPs’ autonomy can later develop. By establishing a safe learning environment through a relation based on trust, instructors enable clients to experiment, make mistakes, and gradually assume more responsibility for their own navigation decisions. Hence, the trust that is established between the instructor and the client is essential because it helps create emotional safety, which would facilitate the learning process in the first place [38,39].

Together, our findings suggest that O&M instructors strive to strengthen all three dimensions of trust, as well-designed O&M training not only supports navigational skill acquisition itself but also the psychological foundation of confidence necessary for independent mobility. Indeed, findings from Bandukda et al. [15] also indicate that O&M training that allows iterative practice enhances self-efficacy by enabling learners to experience success, build confidence, and progressively internalise navigational competencies. Nevertheless, our findings suggest that it might be complex to achieve these goals in practice. The stories of the O&M instructors demonstrate that they are often actively trying to determine what the best approach for a certain client might be (given their personal contextual characteristics and capacities and limits). At each point along a route, they assess when to intervene for safety purposes and when to let the client act autonomously for learning purposes. These instructional decisions can have positive or adverse effects on trust. Intervening might consolidate trust within the instructor–client relationship, but it might also undermine it when a client feels coddled. Not intervening might lead to an increase in the client’s levels of self-confidence and self-efficacy in case of success experiences, but to a decrease in case of failure. From the perspective of the O&M instructors, these decisions can impact training outcomes in consequential ways. Sometimes it might even lead to the cessation of the training trajectory, for example, when a client’s willingness and motivation to learn has dwindled due to a decrease in self-confidence and self-efficacy.

Finally, we speculate that trust is interlinked with individual differences in navigation-related cognitive capacities. Specifically, a combination of a client’s high self-confidence and enhanced self-efficacy, and a safe learning environment brought about by the interpersonal dynamics between the instructor and the client might cultivate a sense of calm required for effective spatial awareness. When individuals feel secure, they can allocate more mental resources to noticing and interpreting relevant environmental cues, for example, auditory information that supports orientation. Conversely, studies with sighted participants suggest that worry and arousal might negatively impact the storage of spatial information in working memory [40,41] and that wayfinding anxiety in specific seems to negatively impact a range of spatial abilities [42,43,44] (though, see also [35] and [45] for counterevidence). Moreover, our data suggest that a higher level of worry in visually impaired individuals could result in an increase in unfavourable behaviours, such as fixating on the immediate walking surface, thereby reducing the capacity to process spatial information in a broader sense. In this way, trust, in all three dimensions, not only enhances a client’s emotional well-being but also contributes directly to the perceptual and cognitive processes necessary for successful independent navigation.

### 4.2. Limitations

Although the diversity of our sample in age, institutional affiliation, geographic location, and years of professional experience enabled us to capture a broad and representative overview of O&M instructors’ perspectives, a limitation of the current study is that our study solely involved Dutch O&M instructors. So, the findings should be interpreted in light of the Dutch welfare system. In the Netherlands, O&M training is reimbursed by the health insurer when people obtained a referral from an ophthalmologist, and it is delivered through specialised national rehabilitation organisations. This reduces potential financial barriers for visually impaired individuals to participate in or complete O&M training. It might also allow for longer training trajectories with a focus on long-term skill development, rather than quicker, outcome-driven training. Such systemic factors may therefore influence both instructors’ training approaches and clients’ expectations, and may differ from countries with other funding models or rehabilitation infrastructures.

Another limitation of the current study is that the interview protocol mainly focused on individual differences regarding personal characteristics and sensory cue use throughout O&M training. Therefore, our study does not present an exhaustive account of all individual differences considered by O&M instructors during training. Nonetheless, our analysis identified several key factors that influence why certain personal characteristics receive particular attention from instructors, for example, safety, trust, and a client’s lifestyle. We also observed variation among instructors in their approaches, including the extent to which they intervene versus allow independent exploration, the emphasis placed on sensory modalities beyond touch and cane use, their openness toward incorporating technological aids, the value they assign to the instructor–client relationship, and the degree to which they explicitly apply the systematic approach of notice-interpret-act-anticipate sensory cues or the ‘completing the sensory picture’ strategy. Understanding both the key reasons that guide these instructional decisions and the diversity of professional perspectives may also inform future hypotheses regarding additional relevant factors in other cultural or institutional contexts not covered by the present study. That is, factors such as safety, trust, and lifestyle influence instructional strategies, although which safety or lifestyle aspects to consider will presumably vary between cultures. Future cross-national comparative studies might provide insight into how relevant individual differences between clients and instructors’ training approaches might vary exactly between countries.

### 4.3. Future Directions

Our findings offer an empirically grounded basis for improving consistency and personalisation in O&M instruction. Currently, O&M instructors rely on their professional expertise to gauge their clients’ sensory skills and preferred sensory modalities during navigation. Yet, several instructors in our study acknowledged that such assessments are not always accurate, and instructional approaches might vary considerably between instructors. These findings point towards the importance of a more standardised method of determining a client’s wayfinding preferences. To address this, findings from the current study and Blokland et al. [2] will be combined to develop a survey instrument that generates a personal sensory navigation profile. Completed prior to the first training session, the profile will capture clients’ subjective sensory preferences and wayfinding tendencies. When integrated with behavioural data, it may help instructors understand how these preferences relate to actual navigational behaviour. This, in turn, could inform decisions about route design, cue selection, and instructional strategies, ensuring that training strategies are better aligned with clients’ perceptual strengths and preferences. Moreover, beyond its initial purpose of informing instructors at the start of the O&M training trajectory, the survey could also serve as a monitoring tool to determine how a client’s focus and self-evaluation may change during the course of the trajectory.

## 5. Conclusions

Although it has been established that individual differences between clients need to be taken into account during O&M training, the current study is the first study to provide an extensive, integrated, and more detailed overview of which individual characteristics matter, how they interact, and how they shape O&M approaches from the perspectives of O&M instructors. We described how route learning for visually impaired clients generally seems to follow a four-step structure (i.e., teaching a client how to notice, interpret, act upon, and anticipate relevant sensory cues until a destination is reached), and that this route learning process is influenced by individual differences in sensory abilities (even fluctuating within a given sensory modality), mental and physical capacities (e.g., concentration, memory, energy levels), and personal characteristics (especially age and anxiety levels). We further state that the dynamic social context in which training takes place is shaped by individual differences between both clients and instructors, which is reflected in, for example, social interactions in context of navigation with either loved-ones, acquaintances, or passersby, or the quality of the instructor–client relationship. Finally, we speculate that themes relating to trust (e.g., building confidence) explain why instructors adapt their training approach according to the reported individual differences, as these themes are associated by the instructors with training outcomes (e.g., attitude towards independent navigation, or speed and success of the actual route learning process). In sum, the stories of our participants provide a unique insight into the personal training approaches of O&M instructors on the basis of which we generated a comprehensive description of the personal, embodied, and social context in which route learning with a visual impairment takes place. Future studies might provide further insight into how training approaches vary across cultures, as well as translate the findings into evidence-based instruments that can be used in real-life training practice to further improve training outcomes and clients’ quality of life.

## Figures and Tables

**Table 1:** Overview of the demographics of our O&M instructor sample. All information is self-reported. Affiliations are anonymised due to privacy (A, B and C). N/A indicates that information was not available.

Participant	Age Range	Level of Education	Affiliation	Experience as O&M Instructor (in Years)	Frequency of Mobility Instruction (Number of Training Sessions a Year)	Predominant Working Area (Degree of Urbanisation According to the Surrounding Address Density)
**01**	50–60	N/A	A	Between 10–15	N/A	N/A
**02**	30–40	Higher professional education (Dutch HBO)	B	Between 5–10	N/A	Extremely urbanised
**03**	40–50	Higher professional education (Dutch HBO)	B	Between 20–25	Around 200–300	Extremely urbanised
**04**	50–60	N/A	A	Between 5–10	N/A	Not to hardly urbanised with some moderately to strongly urbanised areas
**05**	50–60	Higher professional education (Dutch HBO)	B	Between 20–25	N/A	N/A
**06**	60–70	Secondary vocational education (Dutch MBO)	A	Between 35–40	Around 200	Strongly urbanised
**07**	20–30	Higher professional education (Dutch HBO)	B	Between 0–5	Around 150	Strongly to extremely urbanised
**08**	20–30	Higher professional education (Dutch HBO)	C	Between 0–5	Around 100	Working area encompasses all degrees of urbanisation
**09**	40–50	Higher professional education (Dutch HBO)	C	Between 5–10	Around 25	Not to hardly urbanised with some moderately to extremely urbanised areas
**10**	30–40	Higher professional education (Dutch HBO)	C	Between 5–10	Around 100–200	Hardly to strongly urbanised

## Data Availability

The data presented in this study are available on request from the corresponding author due to privacy.

## Abbreviations

The following abbreviations are used in this manuscript:

VIP	Visually impaired person(s)
O&M	Orientation and mobility

## Appendix A

*Interview Protocol*

Instructions and consent

Thank you very much for taking the time participate in this study. Today, I will interview you about your experience pertaining to Orientation and Mobility training. [Briefly introduce yourself and the rest of the research team.]

It has been mentioned in the information letter that it is expedient to record the conversation, so that we can stay as close as possible to what people have said in the investigation. Do you mind if the conversation will be recorded in a moment?

- [If not:] I have a consent form here that states what you are consenting to when you participate in the study, and you can give your consent by putting your name, today’s date, and your signature on the form. Would you please go through the form by yourself? If there are any questions or comments, please let me know.
- [If so:] Would you mind if I wrote down your answers instead of recording them?
  ○ [If not: Write the answers as well as possible and continue with the text below.]
  ○ [If so: Conduct the interview without recording and note-taking, and take notes from memory as soon as possible afterwards.]

Thank you very much. Then we can start in a second. But first I want to emphasize that there are no right or wrong answers in this interview. We are here because you have expertise and important knowledge that can help science, people with visual impairments and other mobility trainers. We are curious about your experiences with the training sessions, and you can mention things that are very obvious to you. Sometimes I will make some notes, that’s for myself for when I start analysing all the interviews, for example when I get an idea for the analysis or hear something that reminds me of something else. This does not mean that the answer was right or wrong.

Do you have any questions or comments before we start the interview?

- [If not:] If there are any questions, you can ask them at any time during or after the interview.

Then I will turn on the audio recorder and we can start! Are you ready?

Introductory questions

1. Could you start by telling us something about yourself—who are you, what do you do in your daily life?
  - Being an instructor during mobility training: when did you start doing that?/How did it come about that you are involved in it now?
2. Thank you for elaborating on that! We will talk about that in detail in the next hour. Have you participated in an interview before?
  - [If so:] Do you remember what kind of questions you were asked then?
  - [If not: Go to question 3.]

Main questions

3. During this interview, I want to talk to you about your experiences with mobility training, and more specifically your observations about the people who follow the training. Can you tell me what a first training session looks like with a client?
4. Can you take me through what the preparation for the first session of a mobility training looks like?
  [Possible follow-up question(s) if it has not yet been discussed:]
  - Which characteristics of the route do you take with you during your preparation with regard to route walking?
  - Which characteristics of the client do you take with you during your preparation for route walking?
5. I would like to talk more about the part of the training where you go outside with the participant to practice route following. Can you think of a client with whom you have recently done a training session for the first time?
  - [If yes:] Would you like to describe this person? [Note whether they mention the visual symptoms, but otherwise everything they mention here may be relevant.]
  - [If not: Continue to question 9.]
  [Possible follow-up question(s):]
  - Are there any other characteristics you pay attention to?
6. Could you take me on the route you walked then, from front door to destination?
7. Which landmarks did you choose so that the client could follow that route?
  [Possible follow-up question(s) if it has not yet been discussed:]
  - Is that something you would do differently for other clients?
  - Do you observe that skill in other clients too?
  - Would you say that that was particularly hard or easy for this client?
8. Are there moments on the route where the client uses multiple senses to maintain the correct route?
9. What feedback, in behaviour or in statements, from the participant did you pay attention to while walking this route?
  - [Refer back to examples from the answer to question 6 when applicable.]
  - Are there certain things that you hear more often from participants? For example, comments about how they used previous training sessions in their daily lives, or about what they found useful and not useful about the training(s)?
10. If a client is heading in the wrong direction, how do you notice that the client themselves realises that they missed their point of reference?
  - What signals do you indicate that that client could pay attention to?
  - When do you intervene?
11. Through which behaviour or statements do you assess the participant’s progress between two sessions?

[If there is time left: Repeat questions 5–9 (partially) for another client with whom the instructor is already familiar.]

Concluding questions

12. An hour has passed, and that means that we have come to the end of the interview. Are there any things you think we missed, or are there other things you would like to mention?

Close

[Provide a summary of the main points talked about and of the central data gathered.]

Then I now turn off the audio recording and would like to thank you very much for your time and participation! We will make an anonymized text file of the audio recording and then analyse it together with the text files from other interviews to see if we can detect certain patterns.

Are there any other things about the research, e.g., that are not about the content that you still want to say?

How did you experience the interview?

[End of protocol.]
